# Development of a New High-Cell Density Fermentation Strategy for Enhanced Production of a Fungus β-Glucosidase in *Pichia pastoris*

**DOI:** 10.3389/fmicb.2020.01988

**Published:** 2020-08-21

**Authors:** Wancang Liu, Haibo Xiang, Tao Zhang, Xu Pang, Jing Su, Hongyu Liu, Baiping Ma, Liyan Yu

**Affiliations:** ^1^Institute of Medicinal Biotechnology, Chinese Academy of Medical Sciences & Peking Union Medical College, Beijing, China; ^2^State Key Laboratory of Biocatalysis and Enzyme Engineering, School of Life Sciences, Hubei University, Wuhan, China; ^3^Beijing Institute of Radiation Medicine, Beijing, China

**Keywords:** *Pichia pastoris*, induction strategy, methanol metabolism, energy generation, biotransformation, high-cell density fermentation, diosgenin

## Abstract

Traditional diosgenin manufacturing process has led to serious environmental contamination and wastewater. Clean processes are needed that can alternate the diosgenin production. The β-glucosidase FBG1, cloned from *Fusarium* sp. CPCC 400709, can biotransform trillin and produce diosgenin. In this study, *Pichia pastoris* production of recombinant FBG1 was implemented to investigate various conventional methanol induction strategies, mainly including DO-stat (constant induction DO), μ-stat (constant exponential feeding rate) and m-stat (constant methanol concentration). The new co-stat strategy combining μ-stat and m-stat strategies was then developed for enhanced FBG1 production during fed-batch high-cell density fermentation on methanol. The fermentation process was characterized with respect to cell growth, methanol consumption, FBG1 production and methanol metabolism. It was found that large amounts of formaldehyde were released by the enhanced dissimilation pathway when the co-stat strategy was implemented, and therefore the energy generation was enhanced because of improved methanol metabolism. Using co-stat feeding, the highest volumetric activity reached ∼89 × 10^4^ U/L, with the maximum specific activity of ∼90 × 10^2^ U/g. After 108 h induction, the highest volumetric production reached ∼403 mg/L, which was ∼91, 154, and 183 mg/L higher than the maximal production obtained at m-stat, μ-stat, and DO-stat strategies, respectively. FBG1 is the first *P. pastoris* produced recombinant enzyme for diosgenin production through the biotransformation of trillin. Moreover, this newly developed co-stat induction strategy represents the highest expression of FBG1 in *P. pastoris*, and the strategy can be used to produce FBG1 from similar *Pichia* strains harboring *Fbg1* gene, which lays solid foundation for clean and sustainable production of diosgenin. The current work provides unique information on cell growth, substrate metabolism and protein biosynthesis for enhanced β-glucosidase production using a *P. pastoris* strain under controlled fermentation conditions. This information may be applicable for expression of similar proteins from *P. pastoris* strains.

## Introduction

Extensive studies have been carried out to find suitable production systems for high-level expression of recombinant proteins (Kita et al., 2016; Rosano et al., 2019; Rozov and Deineko, 2019; Ahmad et al., 2020). The yeast *Pichia pastoris* (*Komagataella phaffii*) has become one of the most widely used expression systems to express several hundreds of proteins from viruses, bacteria, fungi, animals, plants, and humans (Mattanovich et al., 2017; Liu et al., 2019a). As a methylotrophic yeast with eukaryotic machinery, *P. pastoris* can grow on the only carbon source of methanol. The genes are integrated in the genome under the tight regulation of the alcohol oxidase I (*AOX1*) promoter, which leads to exogenous over-expression of recombinant proteins in *P. pastoris*. The protein yields are commonly in the range of milligrams to grams per liter of culture, and claims up to 20–30 g/L recombinant protein (Çelik and Çalık, 2012; Kazemali et al., 2016). Using optimal methanol induction strategies, the yields of heterologous proteins could reach as high as 22 g/L for intracellular production of the recombinant hydroxynitrile lyase (Hasslacher et al., 1997), and 14.8 g/L for extracellular production of the recombinant gelatins (Werten et al., 1999). Additional attributes including high-cell density fermentation on defined media (>100 g/L dry cell weight, or >400 g/L wet cell weight), disulfide bond formation, extracellular secretion of protein, post-translational modifications, and easy scale-up of the bioprocess are other key drivers of *P. pastoris* for industrial purposes (Liu et al., 2016; Ueira-Vieira et al., 2017). In particular, this microorganism was found to be especially effective in producing hard to express proteins, such as membrane proteins and some of eukaryotic glycoside hydrolases, which are expressed poorly in other systems (He et al., 2014; Sajitha et al., 2015; Rueda et al., 2016).

Diosgenin, an important isospirostane-type plant secondary metabolite, has been used extensively in the pharmaceutical industry as a well-known precursor for the synthesis of steroidal drugs (Mohammadi et al., 2019). In industry, diosgenin is prepared mainly by traditional acid hydrolysis (Wang et al., 2014). However, this method causes degradation of diosgenin, and generates a large pollution footprint because of the high concentrations of chemical oxygen demand, sulfate ion, acid, and total nitrogen in wastewater (Zhao et al., 2008; Cheng et al., 2009). We previously found that the fungal *Fusarium* sp. CPCC 400709 could efficiently biotransform steroidal saponins in *Dioscorea zingiberensis* C. H. Wright, and yielded diosgenin in a clean and sustainable process without environmental contamination and waste water (Xiang et al., 2018). The β-glucosidase FBG1, one of the native glycoside hydrolases purified from CPCC 400709, is able to biotransform trillin (diosgenin glucoside) into diosgenin (Supplementary Figure S1), which provides an environmental friendly process to produce diosgenin with an epoch-making significance, and therefore completely innovates the production process for diosgenin. FBG1 belongs to the GH1 family of glycoside hydrolases. Then, the gene *Fbg1* encoding FBG1 was cloned from CPCC 400709, and cloned into pET-30a(+). The resulting plasmid was transformed into *Escherichia coli* Transetta (DE3). We found that very little FBG1 was detected in supernatant of the *E. coli* culture, and primary stored in cell debris in the form of inclusion body (Supplementary Figure S2). Due to the steric effect, *C*-3 glucosyl of trillin is far more difficult to hydrolyse than other steroidal saponins (Feng et al., 2007a, b). In the current study, the *P. pastoris* expression system was employed to produce FBG1 for pharmaceutical purposes. To the best of our knowledge, FBG1 is not only the first enzyme from *P. pastoris* used to efficiently biotransform trillin, but also the first *P. pastoris* produced recombinant enzyme for sustainable diosgenin preparation.

The methanol metabolism for *P. pastoris* is initiated with the oxidization by AOX to formaldehyde and hydrogen peroxide (Jia et al., 2017), both are highly toxic compounds (Resina et al., 2004; Vanz et al., 2012). As shown in Supplementary Figure S3, a portion of the formaldehyde generated from methanol leaves peroxisome and it is oxidized via formate to carbon dioxide by the NAD^+^-dependent formaldehyde dehydrogenase (FLD) and NAD^+^-dependent formate dehydrogenase (FDH) (dissimilation pathway A) (Vanz et al., 2012). These reactions generate reducing power in the form of NADH, thereby serving as a primary source of energy during growth on methanol (Baerends et al., 2002). The remaining formaldehyde is directly fixed with xylulose 5-phosphate to produce glyceraldehyde 3-phosphate, and enters the cell biosynthetic pathway (assimilation pathway B). The assimilation reaction products are used to form cell skeleton and synthesize the targeted protein by a cyclic pathway that starts with condensation of formaldehyde (Gao et al., 2012; Maghsoudi et al., 2012; Fam et al., 2017). Normally, under sole methanol induction the majorities of reductive power NADH for ATP regeneration are originated from dissimilation pathway. TCA cycle also generates NADH, but under methylotrophic metabolism environment this pathway is not essential for energizing proteins but mainly serves for biosynthetic purpose (Yu et al., 2013). Methanol not only is the inducer of *AOX1* promotor and the only carbon source for production, but also the yeast is sensitive to methanol concentration. In general, methanol concentrations can be controlled between 2 and 3.5 g/L (Surribas et al., 2003; Schenk et al., 2007; Potvin et al., 2012). However, induction of *AOX1* promoter by methanol may lead to *Pichia* cell growth inhibition when methanol concentration is over 4 g/L (Potvin et al., 2012; Çelik and Çalık, 2012). The optimal methanol concentration for *P. pastoris* depends on the specific protein, and methanol needs to be added continuously into the fermentation culture with selected methanol induction strategy. Several induction strategies have been established, such as DO-stat, μ-stat, m-stat, etc. (Trinh et al., 2003; Fang et al., 2014; Kastilan et al., 2017). The DO-stat strategy is an indirect feedback mode which automatically controls the substrate feeding rate to efficiently maintain the on-line DO at a near-constant level. The μ-stat strategy is implemented using an open-loop control or the preprogrammed exponential feeding, where a feeding rate profile derived from mass balance equations is employed, and growth rate is theoretically maintained at a constant set-value. The m-stat is created on the basis of maintained constant methanol concentration in the fermentation broth, and is also named methanol non-limited fed-batch strategy. In general, the methanol addition depends on the specific methanol feeding strategy, however, the microorganism itself controls the amount of methanol consumed as a response to its own metabolism, and its concentration is variable. It is likely that the methanol metabolism, cell density, enzyme activity and production yield would depend on the methanol induction strategy (Trinh et al., 2003; Güneş et al., 2016; Nieto-Taype et al., 2020). It is therefore essential to evaluate the various strategies that can be employed in the production process of a recombinant protein. In view of the industrial application prospect of FBG1 for diosgenin production, it is fundamental to explore the best methanol induction strategy to produce active enzyme.

In this work, based on the investigation of three conventional fed-batch methanol induction strategies for *P. pastoris*, mainly including DO-stat (constant induction DO), μ-stat (constant specific cell growth rate), and m-stat (constant methanol concentration), a new combined fed-batch high-cell density fermentation strategy (co-stat strategy) was further developed for enhanced FBG1 production. The co-stat strategy was composed with μ-stat and m-stat strategies. To our best knowledge, studies on fed-batch methanol induction strategies for the production of β-glucosidase implemented to generate diosgenin through recombinant enzyme catalysis are very limited, although various methanol induction strategies have been widely proposed (Potvin et al., 2012; Byrne, 2015; Looser et al., 2015). The present work describes the first-ever attempt to investigate the effect of methanol induction strategies on over-expression of FBG1 in a *P. pastoris* Mut^+^ (methanol utilization plus) strain under controlled fermentation conditions, and represents the highest FBG1 production. Moreover, the co-stat strategy can be used to produce FBG1 from other *Pichia* strains harboring *Fbg1* gene, which lays solid foundation for clean and sustainable production of diosgenin.

## Materials and Methods

### Strains, Plasmids, Media and Chemicals

*Pichia pastoris* GS115 and pPIC3.5K were purchased from Invitrogen Inc. (San Diego, CA, United States). *E. coli* DH5α and pET30a (+) were purchased from Novagen Inc. (Madison, WI, United States). The GS115-ZA-*Pgp* strain containing the sequence encoding P-glycoprotein was constructed previously (Esser et al., 2016). All the plasmids and strains were stored in our laboratory. Media including Luria-Bertani (LB), yeast extract peptone dextrose (YPD), minimal dextrose (MD), minimal methanol (MM), buffered glycerol complex (BMGY), buffered methanol complex (BMMY), basal salts medium (BSM), and *Pichia* trace metal (PTM) solution were prepared as per the Invitrogen’s multi-copy *Pichia* expression kit. Plasmid midi, PCR purification, and gel extraction kits were purchased from Qiagen Inc. (Chatsworth, CA, United States). The restriction enzymes, phusion high-fidelity DNA polymerase and T4 DNA ligase were obtained from New England Biolabs Inc. (Ipswich, MA, United States). *p*-Nitrophenyl-β-D-glucopyranoside (*p*NPG) was purchased through Sigma-Aldrich Inc. (St. Louis, MO, United States). Other chemicals were obtained from Thermo Fisher Scientific (Waltham, MA, United States). All other reagents used were of reagent grade. DNA primers synthesis and capillary DNA sequencing were performed by Center for Biologics Evaluation and Research (CBER of FDA, Silver Spring, MD, United States).

### Plasmid Construction and *P. pastoris* Transformation

The plasmid pET30a-*Fbg1* carrying a cDNA sequence of the mature FBG1 was used as template in PCR with forward primer 5′-CCGGAATTCATGACACCCTCACACGCTGTGATAC-3′ (*EcoR* I site on the 5′ end is underlined) and reverse primer 5′-A T A A G A A TG C G G C C G CC T C CACACCACGCGCCTTC-3′ (*Not I* site on the 5′ end is underlined) involving initial denaturation at 98°C (30 s), 35 cycles of amplification at 98°C (10 s), 55°C (30 s), and 72°C (1 min and 15 s), followed by a final extension at 72°C (10 min). Both the PCR product and the plasmid pPIC3.5K were digested with *EcoR* I and *Not* I. After gel-purification, the fragments were ligated at 16°C overnight. *E. coli* DH5α was transformed with the resulting expression plasmid pPIC3.5K-*Fbg1* and isolated plasmids from single colony transformants, selected on LB-agar plates containing 100 mg/mL ampicillin, were analyzed for correct orientation of the inserted gene. The sequence of pPIC3.5K-*Fbg1* was verified by capillary DNA sequencing using forward primers 5′-GACTGGTTCCAATTGACAAGC-3′ (5′AOX) and reverse primer 5′-GCAAATGGCATTCTGACATCC-3′ (3′AOX).

With slight modifications, the *P. pastoris* transformation was carried out as per instruction of “Invitrogen’s multi-copy *Pichia* expression kit” and “Invitrogen’s pPIC3.5K/pAO815 *Pichia* vectors for multi-copy integration and intracellular expression.” In brief, ∼10 μg purified DNA fragment of pPIC3.5K-*Fbg1* was linearized by *Sac* I and transformed into 50 μL overnight grown *P. pastoris* GS115 cells (∼0.8 optical cell density at 600 nm, OD_600_) prepared using standard methods by the lithium chloride transformation method (Li and Xia, 2018). The parent pPIC3.5K vector linearized in the same manner was used as a control. Resulting yeast cells were gently suspended in 1.0 mL sterile water, and then selected on MD and MH-agar plates after incubation for ∼72 h at 28°C. Using the post-transformational vector amplification method (plate PTVA) (Sunga et al., 2008), the transformants were further selected using YPD-agar plates containing increasing concentrations of G418 with the final concentrations of 0.5, 1.0, 1.5, 2.0, and 2.5 mg/mL. Recombinant clones were confirmed by PCR using gene specific primers.

### Screening of Transformants

Transformants selected from YPD-agar plates containing 2.5 mg/mL G418 were grown in shake flasks without baffle for further screening. Meanwhile, about twenty-five transformants selected randomly from YPD-agar plates containing 0.1, 0.5, 1.0, 1.5, and 2.0 mg/mL G418 (five transformants for each concentration) were also comparatively investigated. Selected transformants could grow on plates containing lower G418 concentrations, but could not grow on plates containing higher G418 concentrations. Briefly, transformants were grown in 25 mL BMGY at 28°C, 280 rpm in 125 mL shake flasks to a final OD_600_ of 5.0. Shake flasks were placed in a thermostatic shaker (New Brunswick Innova 44R, Enfield, CT, United States). Cells were then harvested by centrifugation at 3,000 × *g*, 28°C for 5 min and resuspended in 25 mL BMMY. 1% (*v/v*) methanol was added into the culture every day to activate the *AOX1* promoter and produce FBG1 at 28°C, 280 rpm for 120 h. For analytical assays, 4 mL sample was withdrawn from 25 mL culture every day. The rest culture was discarded, and next-day sample was collected from other shake flasks. Transformant exhibiting the highest activity was picked and cryopreserved at −80°C. Before each experiment, YPD-agar plate containing 2.5 mg/mL G418 was used to activate the selected positive transformant.

### High-Cell Density Fermentation Process

Bench-top high-cell density fermentation of the selected *P. pastoris* strain was studied in 5 L fermenters (Sartorius Stedim Biotech, Göttingen, Alemania, Germany). The fermenters were interfaced to AFS-Biocommand bioprocessing software and Sartorius MFCS/win 3.0 system. The fermenters were also equipped with a methanol probe with sensor unit MRK002 from Raven Biotech Inc. (Vancouver, BC, Canada) and a feeding pump 101U/R from Watson-Marlow Limited (Falmouth, Cornwall, United Kingdom). A single colony of the chosen strain from YPD-agar plate containing 2.5 mg/mL G418 was inoculated in a 1 L shake flask (without baffle) containing 300 mL BMGY medium. The culture was grown overnight at 28°C in a shaking incubator at 260 rpm to expand cultivation until the OD_600_ was 6 (about 20–24 h). Then, this culture was inoculated in 3 L BSM medium held in a 5 L fermenter with initial OD_600_ of 0.6. Using the standard method reported previously (Liu and Zhu, 2015; Wang et al., 2017), the fermentation operation was divided into three phases: P1, a glycerol batch phase; P2, a glycerol fed-batch phase; and P3, the methanol fed-batch phase. Glycerol fed-batch cultivation was applied to increase the cell density for improved FBG1 production. It was started after ∼20 h of glycerol batch cultivation by feeding in 50% glycerol (*m/m*) solution containing 12 mL/L PTM, which was then added at a rate of 18 mL/h/L. Before starting the methanol fed-batch phase, the glycerol feed was completely stopped for 0.5 h to avoid repression of the *AOX1* promoter and a biomass of 200 OD_600_ was achieved. In the methanol induction phase, pure methanol containing 12 mL/L PTM was supplemented into fermenter through specific induction strategy. The temperature was maintained at 28°C. The pH was kept at 5 by using ∼7.5% ammonia hydroxide solution or ∼20% phosphoric acid. The foam was controlled by adding 0.03% (*v/v*) antifoam reagent of polypropylene glycol P2000 (Fluka, St. Louis, MO, United States) before heat sterilization, and certain amounts of antifoam reagent was added at the induction phase to de-foam. This antifoam regent did not affect cell growth and FBG1 production. Agitation was kept within the range 50–800 rpm. Unless indicated, dissolved oxygen (DO) was maintained at 25% throughout the fermentation. The aeration was manually adjusted from 0.2 to 1.5 *vvm*. Air was always sparged into bioreactor during the entire methanol induction period. Pure oxygen was sparged with air when DO was not be maintained at set point. At the start of methanol induction, fermentation broth samples were taken regularly every 12 h to measure the biomass, protein, and enzyme activity. At the end of fermentation, cell pellets containing crude enzyme obtained through centrifugation at 6,000 rpm for 15 min was collected.

### Fed-Batch Fermentation Strategies

Three conventional strategies (DO-stat, μ-stat, and m-stat strategy) and the newly developed strategy (co-stat strategy) were implemented. (A), strategy based on oxygen consumption (DO-stat, DO = 25%): DO was controlled by a DO cascade of agitation. Methanol feeding was paused when DO value was below 20%, while continued when the DO recovered to above the set point by measuring the concentration of DO at each minute. (B), strategy based on specific cell growth rate (μ-stat, μ = 0.015 h^−l^): After P3, methanol addition was initiated under the predetermined exponential feeding rate. Methanol was automatically added into fermenters using substrate pump. Feeding profile was derived from reported *Pichia* mass balance equation (Zhang et al., 2000). (C), strategy based on methanol consumption (m-stat, methanol concentration = 2 g/L): An on-line methanol probe equipped with sensor unit and a feeding pump to monitor and control methanol concentration in the vessel. The internal regulation software of the sensor was used to control the external peristaltic pump and adjust methanol concentration constantly maintained at the set point. There was no significant difference in slight fluctuation of methanol concentrations was observed. (D), strategy based on combination of μ-stat and m-stat strategies (co-stat strategy): After P3, methanol supplement was separately processed by two combined strategies of μ-stat and m-stat. μ-Stat was performed first and the methanol addition was ramped exponentially with an exponential feeding rate at 0.015 h^–1^. After accumulated residual methanol in the fermentation broth reached 2 g/L, μ-stat process was terminated and the m-stat process was started, where the methanol concentration was maintained at 2 g/L to the end of the harvest. For the three conventional strategies, the on-line DO levels, exponential feeding rates, and maintained methanol concentrations were pre-optimized in 1 L fermenters containing 600 mL media (Sartorius Stedim Biotech, Göttingen, Alemania, Germany). Three levels of each strategy were studied. DO levels for DO-stat strategy: 50, 25, and 10%. Exponential feeding rates for μ-stat strategy: 0.007, 0.015, and 0.030 h^−l^. Methanol concentrations for m-stat strategy: 0.5, 2.0, and 4.5 g/L. All other fermentation conditions for 1 L fermenters were same as that used for 5 L fermenters.

### Analytical Assays and Statistical Analysis

#### Preparation of Cell Lysate

After centrifugation at 12,000 × *g*, 4°C for 3 min, the supernatant and cell pellets from fermentation broth were collected separately and stored at −80°C. The method for preparing cell lysate was similar to that described previously (Suye et al., 1990; Jin et al., 2010). In brief, cell pellets were washed twice with 50 mM phosphate buffer (pH 7.5), re-centrifuged, and then resuspended in the same buffer with the volume ratio of 1:1. After adding same volume of glass beads (diameters were ∼500 μm), the suspension was sonicated by glass beads assisted sonication at 4°C for 25 min (Sonicator XL, Heat system-Ultrasonics Inc., San Jose, CA, United States). Followed by centrifugation at 15,000 rpm, 4°C for 30 min, resulting supernatant was used as cell lysate. The cell lysate was properly diluted to prepare the assay solution before measurement.

#### Quantifications of Methanol and Formaldehyde

As previously reported methods (Liu et al., 2019b), methanol in the fermentation broth was measured with an GM8 Micro-stat Analyzer (Analox Instruments Ltd., Hammersmith, London, United Kingdom). Using a formaldehyde assay kit (Sigma-Aldrich, St. Louis, MO, United States, MAK131) as per manufacturer’s instruction, formaldehyde (CH_2_O) was measured by derivatization with acetoacetanilide in the presence of ammonia. Briefly, the appropriately diluted sample was mixed with 50 μL reaction mix. After incubating at 25°C for 30 min, fluorescence was recorded in a microplate reader (SpectraMAX i3, Molecular Devices, Sunnyvale, CA, United States) with 370 nm excitation and 470 nm emission wavelengths. The concentration of formaldehyde (millimolar formaldehyde per gram dry cells, mM in cells) was normalized by the concentration of biomass (gram dry cells per liter fermentation broth, g/L), and determined from the standard curve. As to formaldehyde standard for fluorometric detection, 5 μL of 10 mM formaldehyde standard was mixed with 495 μL water to create a 100 μM working standard, and then diluted as indicated in the instruction.

#### Activity Assays of Enzymes Related to Methanol Metabolism

Alcohol oxidase (AOX, EC 1.1.3.13) activity was spectrophotometrically measured in the presence of 2,2-azino-bis(3-ethylbenzthiazoline-6-sulpho-nic acid) and horseradish peroxidase (Arrocha et al., 2014). One unit of AOX activity is defined as formation of 1 μmol H_2_O_2_ per minute. The formaldehyde dehydrogenase (FLD, EC 1.2.1.46) and formate dehydrogenase (FDH, EC 1.2.1.2) activities were determined according to the previously described method (Allais et al., 1983; Kato, 1990; Tran et al., 2015). One unit of the enzyme activity was defined as the amount of enzyme catalyzing the formation of 1 μmol NADH per minute.

#### Biomass Measurement

The OD_600_ of the appropriately diluted fermentation broth was determined with UV/VIS spectrophotometer. Cell density (dry cell weight, DCW) was obtained by centrifuging 2 mL samples in the pre-weighed centrifuge tubes at 12,000 × *g* for 5 min and washing twice with distilled water, then allowing the pellets to dry to constant weight with a moisture determination balance (MB 200, Ohaus Corp., Florham Park, NJ, United States) by heating the samples at 95°C for 3 h. Alternatively, DCW was also estimated after correlation DCW with OD_600_. In brief, 2 mL fermentation broth was withdrawn at six different OD_600_ values varied among 200–400. After OD_600_ measurement, cell pellets were brought to moisture determination balance for DCW determination. Then, DCW was estimated according to the following equation: DCW = OD_600_ × dilution factor × 0.27 (Eq. 1).

#### β-Glucosidase Activity Assay

A standard colorimetric assay was developed on the basis of previously reported methods with slight modifications (Liu and Zhu, 2015; Kornecki et al., 2020; Wang et al., 2020). In brief, 25 μL appropriately diluted fermentation broth (containing cells) was added to 75 μL of 5 mM *p*NPG. After incubation at 50°C for 20 min, 300 μL saturated Na_2_B_4_O_7_ solution was added to terminate the enzymatic reaction. The β-glucosidase activity was determined on the basis of absorbance at 405 nm, and then evaluated by both volumetric activity (U/L) and specific activity (U/g). The enzymatic activity of parental strain GS115 was assayed in the same manner, and used as a control. The average β-glucosidase activity of the control sample from entire methanol induction period was deducted before calculating the enzymatic activity of FBG1. One unit of enzyme activity was defined as the amount of enzyme required to release one nM *p*-nitrophenol from *p*NPG per minute at 50°C and pH 5.

#### SDS-PAGE Analysis and FBG1 Determination

SDS-PAGE was performed based on previously reported methods with some modifications (Liu et al., 2016, 2019b). Briefly, 0.5 g freeze-dried yeast cells were suspended in 5 mL 50 mM phosphate buffer containing 1 mM PSMF (pH 7.5) and subjected to 25 cycles of glass beads assisted cell disruption at 4°C for 25 min. The supernatant was obtained by centrifugation at 15,000 rpm, 4°C for 30 min. After gradient elution of different concentration of imidazole by nickel bonded affinity chromatography, fractions eluted from buffer containing 60 mM imidazole were pooled and concentrated by ultrafiltration (Amicon Ultra-15, UFC901096 10KD, Millipore, Bedford, MA, United States). 15 μL resulting sample was mixed with 5 μL 4 × Laemmli Sample Buffer (Bio-Rad Laboratories, Hercules, CA, United States). Then, SDS-PAGE was executed using a NuPAGE 12% Bis-Tris gel (Invitrogen, Carlsbad, CA, United States) on a Mini Gel Tank (Invitrogen, Carlsbad, CA, United States) as previously described (Laemmli, 1970). Protein molecular weight marker was purchased from New England Biolabs Inc. (Ipswich, MA, United States). Gels were stained with SimplyBlue^TM^ SafeStain (Thermo Fisher Scientific, San Jose, CA, United States). The SDS-PAGE band was visualized using an Amersham^TM^ Imager 600 (GE Healthcare, Boston, MA, United States). Using purified FBG1 as a reference, the volumetric production (mg/L) was determined by scanning the area of each SDS-PAGE band, and calculated with ImageQuant TL software (GE Healthcare, Boston, MA, United States) on the basis of concentration factor.

#### Other Calculations and Statistical Analysis

Other calculations including specific cell growth rate (μ), specific methanol consumption rate (g/g/h), volumetric FBG1 production (mg/L), specific FBG1 production (mg/g), and specific production rate (mg/g/h) were determined using described methods (Liu et al., 2016, 2019b). Experiments were done in triplicates. For each data, replicates from three parallel measurements or independent assays were measured and the mean ± standard error (S.D.) was calculated. Student’s *t*-test in SPSS 17.0 (SPSS Inc., NY, United States) was used for two-group comparisons. ^∗^*P* < 0.05 was considered statistically significant.

## Results

### Construction and Screening of the Engineered Yeast

Using a pair of custom-designed primers, the desired fragment of *Fbg1* gene was amplified by PCR, which showed the expected size of 1,863 bp (Supplementary Figure S4). The *Fbg1* gene without codon optimization was then sub-cloned into a pPIC3.5K vector, and PCR-amplified fragments from transformants grown on LB plates containing 100 μg/mL ampicillin showed the predicted size of ∼2,000 bp. The capillary DNA sequencing result showed that the sequence of *Fbg1* gene was correct. After construction, the resulting plasmid of pPIC3.5K-*Fbg1* was transformed into *P. pastoris* GS115 and seeded on YPD-G418-agar plate. Colonies grown on the plate with low-G418 concentration were again spread on YPD-agar plates containing a large amount of G418 with the highest concentration of 2.5 mg/mL.

Later, the recombinant yeast transformants grown on YPD-agar plates containing various G418 concentration were cultured and induced for FBG1 expression in shake flasks. According to β-glucosidase activity assay of selected *P. pastoris* transformants, all showed intracellular expression of FBG1 (Table 1). Strain GS115-3.5K-*Fbg1*-s3 demonstrated the highest β-glucosidase activity at the induction time of 120 h with the value of 11.6 × 10^4^ U/L (Supplementary Figure S4). Due to the glycosylation of post-translational modifications in *P. pastoris*, *Pichia* produced FBG1 shows protein band ∼95 kDa, which is ∼20 kDa larger than the molecular weight of *E. coli* produced FBG1 (Supplementary Figure S2). There was no protein expression observed at the predicted molecular weight from the empty pPIC3.5K plasmid and the parental strain GS115. Because it showed the highest level of FBG1 expression, the recombinant GS115-3.5K-*Fbg1*-s3 was used for further experiments.

**TABLE 1:** β-Glucosidase activities of selected *P. pastoris* GS115 transformants in shake flasks.

**Selected transformants**	**β-Glucosidase activities (U/L × 10^4^)**	**Average enzymatic activity (U/L × 10^4^)**	**G418 concentrations (mg/mL)**
s1	10.8 ± 0.3	10.7 ± 0.9 (*n* = 3)	2.5
s2	9.8 ± 0.2		
s3	11.6 ± 0.3		
s4	9.7 ± 0.3	9.5 ± 0.9 (*n* = 5)	2.0
s5	10.4 ± 0.2		
s6	10.1 ± 0.2		
s7	8.2 ± 0.4		
s8	9.1 ± 0.2		
s9	7.1 ± 0.2	7.5 ± 0.6 (*n* = 5)	1.5
s10	8.3 ± 0.3		
s11	6.9 ± 0.3		
s12	7.5 ± 0.1		
s13	7.8 ± 0.2		
s14	6.1 ± 0.2	5.2 ± 0.9 (*n* = 5)	1.0
s15	3.8 ± 0.1		
s16	5.7 ± 0.2		
s17	5.0 ± 0.1		
s18	5.6 ± 0.2		
s19	2.4 ± 0.1	2.9 ± 0.6 (*n* = 5)	0.5
s20	3.1 ± 0.2		
s21	2.9 ± 0.4		
s22	2.7 ± 0.3		
s23	3.6 ± 0.2		
s24	0.77 ± 0.06	0.8 ± 0.2 (*n* = 5)	0.1
s25	0.69 ± 0.05		
s26	1.26 ± 0.3		
s27	0.74 ± 0.1		
s28	0.69 ± 0.06		
GS115	∼0	N.A	0
GS115-3.5K	∼0	N.A	0.1

*Twenty-eight transformants were selected from YPD-agar plates containing 0.1, 0.5, 1.0, 1.5, 2.0, and 2.5 mg/mL G418. GS115 and GS115-3.5K indicate controls of the parental strain GS115 and the strain harboring empty pPIC3.5K vector, respectively. The β-glucosidase activity of the parental strain was deducted before calculating the enzymatic activity of recombinant FBG1. Results reflect the maximal values during entire 120 h induction. Experiments were done in triplicates. Three parallel measurements for each transformant were assayed. Values are indicated by means ± S.D. N.A indicates not available.*

### High-Cell Density Fermentation Process and Pre-optimization

The high-cell density fermentation process was divided into three phases, namely glycerol batch fermentation, glycerol fed-batch fermentation and methanol fed-batch fermentation. Before methanol supplementation, the fermentation was held for 0.5 h without carbon source addition. Fed-batch high-cell density fermentation was performed using conventional strategies (DO-stat, μ-stat, and m-stat strategy) and the newly developed co-stat strategy. Prior to investigation on various methanol induction strategies, the pre-optimization of DO saturation, exponential feeding rate and methanol concentration at induction phase was initially carried out for DO-stat, μ-stat and m-stat strategies, respectively (Supplementary Table S1). DO of 25% demonstrated higher β-glucosidase activity and FBG1 production than that observed at the 10 and 50% DO. Among the three exponential feeding rates, the best FBG1 over-expression was seen at the exponential feeding rate of 0.015 h^−l^. It was found that the process with 2.0 g/L methanol demonstrated higher enzymatic activity and production than that obtained at 0.5 and 4.5 g/L. For the following experiments, DO level, exponential feeding rate and maintained methanol concentration were therefore set as 25%, 0.015 h^−l^ and 2 g/L at the DO-stat, μ-stat and m-stat strategies, respectively. The co-stat strategy was composed with two parts, one part was 40 h μ*-*stat strategy with a constant exponential feeding rate of 0.015 h^−l^, and the following part was 68 h m-stat strategy at a constant 2 g/L methanol concentration.

### Cells Growth During Various Fed-Batch Processes

**FIGURE 1:**
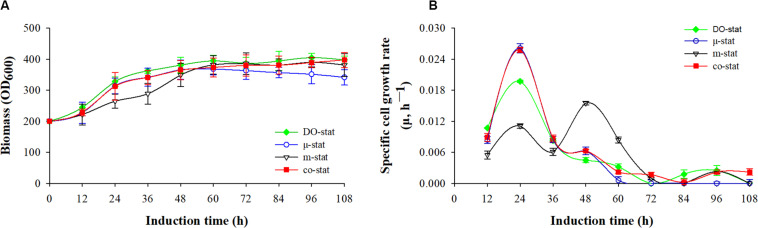
Cell growth in response to different induction strategies. **(A)** Optical cell density (OD_600_). **(B)** specific cell growth rate (μ, h^– 1^). Time zero indicates the initiation of methanol addition. DO-stat strategy (filled green diamond), μ-stat strategy (open blue circle), m-stat strategy (open black triangle), co-stat (filled red square).

Cells growth was characterized with respect to biomass accumulation and specific cell growth rate (Figure 1). Similar cells growth was observed at the initial 36 h induction periods of μ-stat and co-stat strategies. Significant difference in cells growth was observed at the entire 108 h induction periods because of the implementation of different methanol induction strategies.

As shown in Figure 1A, methanol induction was started when the *P. pastoris* cell density reached 200 OD_600_, which was equivalent with 50 g/L dry cell weight (DCW). All the processes were conducted with the same starting induction biomass and harvested after 108 h induction. The maximal OD_600_ reached 405, 397, 390, and 368 when using the DO-stat (at 96 h), co-stat (at 108 h), m-stat (at 96 h), and μ-stat strategy (at 60 h), respectively. However, over 27 OD_600_ decrease was seen at the last 48 h induction period of μ-stat process although 168 OD_600_ increase was observed during the initial 60 h induction period. Conversely, the biomass almost kept constant at the last 48 h induction period of m-stat process. With the combination of μ-stat and m-stat strategies, OD_600_ increased sharply to 313 at the induction time of 24 h, and then continued with an increase of 52 OD_600_ during the following 24 h induction period (365 OD_600_ at 48 h). Meanwhile, slight increase in biomass (∼24 OD_600_) was obtained during the last 48 h induction period of co-stat process, with an average 384 ± 9.3 OD_600_.

Figure 1B shows the specific cell growth rate (μ, h^–1^). DO-stat strategy demonstrated a maximum of 0.020 h^–1^ at the induction time of 24 h, whereas 0.015 h^–1^ decrease was observed during the following 24 h induction period. Using m-stat strategy, the maximal rate was 0.016 h^–1^ (at 48 h), and then the value decreased significantly to 0.001 h^–1^ (at 72 h). Exponential methanol feeding rate was theoretically maintained at set-value of 0.015 h^–1^ at the μ-stat strategy. Unexpectedly, the experimental specific cell growth rate increased rapidly from 0.008 h^–1^ (at 12 h) to the highest rate of 0.026 h^–1^ (at 24 h). Compared with theoretical set-values, 0.009 and 0.013 h^–1^ lower experimental rates were seen at the induction time of 48 and 60 h, respectively. At the μ-stat period of co-stat process, the experimental maximum was also 0.011 h^–1^ higher than the theoretical set-rate. During the last 36 h induction period, the average specific cell growth rates were 0.001 ± 0.001, 0.001 ± 0.001, and 0.002 ± 0.001 h^–1^, respectively for DO-stat, m-stat and co-stat strategy.

### Effect of Various Fed-Batch Processes on the Methanol Consumption

**FIGURE 2:**
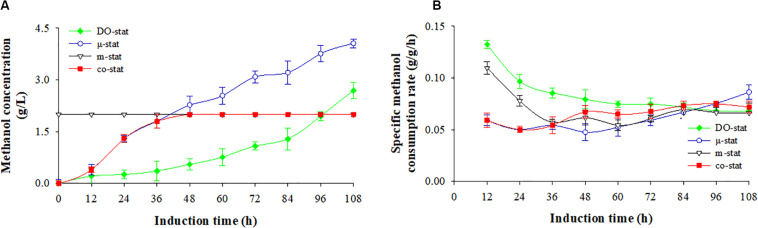
Methanol consumption at four induction strategies. **(A)** Methanol concentration (g/L). **(B)** specific methanol consumption rate (g/g/h). Time zero indicates the initiation of methanol addition. DO-stat strategy (filled green diamond), μ-stat strategy (open blue circle), m-stat strategy (open black triangle), co-stat (filled red square). Methanol concentrations at m-stat process (including the m-stat part at co-stat strategy) indicate the set-values.

Figure 2 shows the methanol consumption by *P. pastoris* producing FBG1, with both the residual methanol concentration in fermentation broth (g/L) and specific methanol consumption rate (g/g/h, dry cells). Using different methanol induction strategy, the varied methanol consumption was followed.

Using DO-stat and μ-stat strategies, methanol was continuously accumulated during the entire induction period (Figure 2A). After 108 h induction, the DO-stat strategy indicated an average methanol concentration of 0.9 ± 0.9 g/L, with a maximum of 2.7 g/L. The average and the highest concentrations at the μ-stat reached 2.2 ± 1.4 and 4.1 g/L (at 108 h), respectively. At the m-stat process, the methanol concentration was controlled and monitored automatically at 2 g/L by an on-line methanol probe immersed in fermentation broth. Although very slight fluctuation took place (*P* < 0.001, *n* = 3), there was no significant difference in methanol concentrations was observed (data not shown). When combining μ-stat and m-stat strategies, the methanol concentration first increased from 0.4 (at 12 h) to 1.8 g/L (at 36 h of μ-stat period), and then remained at theoretical set-value of 2 g/L from the induction time of 40 h to the harvest point (m-stat period).

Figure 2B shows the specific methanol consumption rate. DO-stat demonstrated the highest rate of 0.133 g/g/h (at 12 h), however, the value decreased dramatically to 0.075 g/g/h (at 72 h), and then remained at constant level with an average value of 0.069 ± 0.002 g/g/h. Similarly, the rates decreased sharply from a maximum of 0.110 (at 12 h) to 0.057 g/g/h (at 36 h) at the m-stat process, and followed by a constant level with an average rate of 0.063 ± 0.005 g/g/h. When the co-stat strategy was employed, the specific methanol consumption rate increased from 0.059 (at 12 h) to 0.072 g/g/h (at 108 h) with a maximum of 0.075 g/g/h (at 96 h). During the last 24 h induction period, the rates remained constant with an average value of 0.073 ± 0.002 g/g/h. After entire 108 h induction, the co-stat strategy demonstrated an average rate of 0.065 ± 0.009 g/g/h, which was close to the rates where only μ-stat or m-stat strategy was used. Due to varied methanol induction strategy, significant difference in methanol consumption was observed.

### Glucosidase Activities During Various Fed-Batch Processes

**FIGURE 3:**
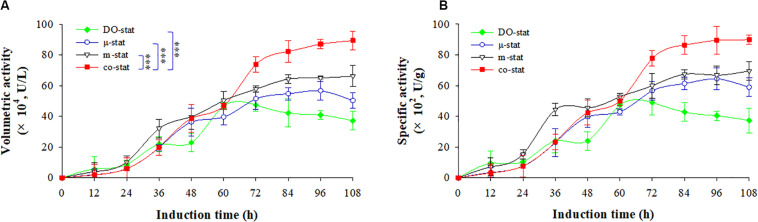
Enzymatic activity at four induction strategies. **(A)** Volumetric activity (U/L). **(B)** specific activity (U/g). Time zero indicates the initiation of methanol addition. DO-stat strategy (filled green diamond), μ-stat strategy (open blue circle), m-stat strategy (open black triangle), co-stat (filled red square). Asterisk indicates the two-group have significant difference (^∗∗∗^*P* < 0.001).

Figure 3 shows the β-glucosidase activities of recombinant FBG1, with both the volumetric activity (U/L) and specific activity (U/g). The background expression of *Pichia* β-glucosidase was deducted by calculating the β-glucosidase activity of parental strain GS115 (Supplementary Figure S5). The highest β-glucosidase activity of FBG1 was obtained at the co-stat process.

Using DO-stat strategy, the maximal volumetric activity was 47.3 × 10^4^ U/L at the induction time of 72 h, which indicated the lowest values while compared with the volumetric activities obtained from other strategies (Figure 3A). Followed by 36 h induction, the volumetric activity decreased significantly with the decrease of 10.2 × 10^4^ U/L (*P* < 0.05, *n* = 3). When the m-stat strategy was used, the maximal volumetric activity reached 66.3 × 10^4^ U/L (at 108 h), which were 9.6 × 10^4^ U/L higher than the maximum obtained at the μ-stat process (at 96 h). Meanwhile, the m-stat process demonstrated constant activity level during the last 24 h induction period with the average value of 65.3 ± 1.0 × 10^4^ U/L.

When the co-stat strategy was implemented, the volumetric activity increased from 1.8 × 10^4^ (at 12 h) to 19.8 × 10^4^ U/L (at 36 h) during the initial μ-stat phase. After the following 36 h induction, the value was up to 73.9 × 10^4^ U/L (at 72 h), which was much higher than that obtained during other processes at this time (*P* < 0.001, *n* = 3). Then, the volumetric activity continuously increased to the highest value of 89.4 × 10^4^ U/L, which was 23.1 × 10^4^ U/L higher than the maximum of the process where only m-stat strategy was used, and 32.7 × 10^4^ U/L higher than the maximum of the only μ-stat feeding process. As a result, consistent specific production was obtained at the co-stat strategy (Figure 3B). After 108 h induction, the highest specific production reached 90.0 × 10^2^ U/g, which was 20.4 × 10^2^ and 25.5 × 10^2^ U/g higher than the maximums of the only m-stat and μ-stat feeding process, respectively. The co-stat strategy exhibited the best performance in terms of β-glucosidase activity of recombinant FBG1.

### FBG1 Production During Various Fed-Batch Processes

Volumetric production of fermentation broth (mg/L), specific production of FBG1 in cells (mg/g, dry cells), and specific production rate (mg/g/h, dry cells) were, respectively, followed during the processes of DO-stat, μ-stat, m-stat and co-stat. The highest volumetric production, specific production, and specific production rate were observed when the co-stat strategy was employed.

**FIGURE 4:**
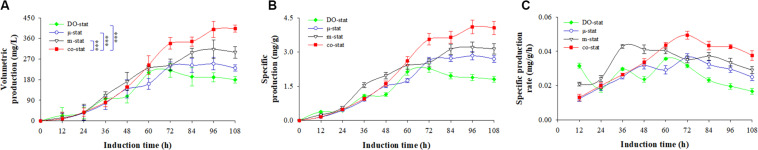
FBG1 production in response to different induction strategies. **(A)** Volumetric production (mg/L). **(B)** specific production (mg/g). **(C)** specific production rate (mg/g/h). Time zero indicates the initiation of methanol addition. DO-stat strategy (filled green diamond), μ-stat strategy (open blue circle), m-stat strategy (open black triangle), co-stat (filled red square). Asterisk indicates the two-group have significant difference (^∗∗∗^*P* < 0.001).

As shown in Figure 4A, the co-stat strategy indicated the highest volumetric production of 403.3 mg/L (at 108 h), which was around 2-fold higher than the maximum obtained at DO-stat process (at 60 h). Production at the co-stat process increased exponentially from 38.2 (at 24 h) to 338.6 mg/L (at 72 h). After 96 h induction, the value increased to 399.0 mg/L, which was 208.2, 149.2, and 86.4 mg/L higher than that obtained at the processes of DO-stat, μ-stat and, m-stat, respectively. During the last 36 h induction period, a 64.7 mg/L increase was observed when the co-stat strategy was employed. By comparison, the m-stat process demonstrated a 55.1 mg/L increase at this period, 40.4 and 9.5 mg/L decreases were observed when DO-stat and μ-stat strategy was implemented, respectively. Resulting specific production is seen in Figure 4B. Compared with volumetric production, similar tendency in the specific production was obtained. The co-stat process shows the highest specific production of 4.1 mg/g at the induction time of 108 h. Using DO-stat, μ-stat, and m-stat feeding strategy, the maximums were 2.3 (at 72 h), 2.8 (at 96 h), and 3.2 (at 96 h) mg/g, respectively, which were 1.8, 1.3, and 0.9 mg/g lower than the highest specific production obtained at the co-stat strategy.

Specific production rate is illustrated in Figure 4C. The co-stat process indicated the highest rate of 0.050 mg/g/h at the induction time of 72 h. After 108 h induction, the entire average rate at co-stat strategy reached 0.034 ± 0.012 mg/g/h, which was 0.009 and 0.027 mg/g/h higher than that obtained during the DO-stat and μ-stat processes, respectively (*P* < 0.05, *n* = 3). When only the μ-stat feeding was used, the maximum was 0.037 mg/g/h (at 72 h). A maximum of 0.043 mg/g/h (at 36 h) was observed when only the m-stat strategy was employed. However, combining μ-stat and m-stat strategies increased the specific production rate from 0.013 (12 h) to 0.044 mg/g/h (60 h). During the last 72 h induction period of co-stat process, the average rate reached 0.040 ± 0.008 mg/g/h, which was much higher than that obtained at the other processes (*P* < 0.001, *n* = 3). This newly developed co-stat strategy gives the most robust performance in FBG1 production.

### Effect of Various Fed-Batch Processes on the Methanol Metabolism

To understand the noticeable differences in FBG1 production at the four methanol induction strategies, enzymes associated with methanol metabolism, such as AOX, FLD, FDH, and methanol metabolite of formaldehyde were then followed for further elucidation of enhanced FBG1 expression in *P. pastoris*. The optimum sonication time was determined (Supplementary Figure S6). After 20–30 min glass beads assisted sonication, the maximal β-glucosidase activity of FBG1 producing strain was observed.

**FIGURE 5:**
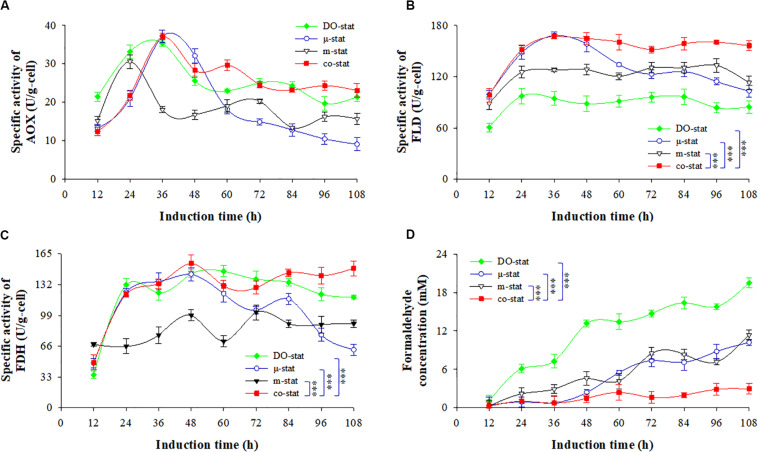
Intracellular activities of AOX, FLD, FDH and formaldehyde concentration at four induction strategies. **(A)** Activity of alcohol oxidase (AOX, U/g-cell). **(B)** activity of formaldehyde dehydrogenase (FLD, U/g-cell). **(C)** activity of formate dehydrogenase (FDH, U/g-cell). **(D)** formaldehyde concentration (mM). Time zero indicates the initiation of methanol addition. DO-stat strategy (filled green diamond), μ-stat strategy (open blue circle), m-stat strategy (open black triangle), co-stat (filled red square). Asterisk indicates the two-group have significant difference (^∗∗∗^*P* < 0.001).

Figure 5A shows the AOX activity. After 36 h induction, the AOX activity at co-stat strategy increased from 12.4 U/g (at 12 h) to the highest activity of 37.1 U/g, which was similar to the maximums obtained at DO-stat and μ-stat processes, but 19.0 U/g higher than that obtained at the m-stat strategy. During the last 60 h induction period, the co-stat strategy demonstrated much higher AOX activities than where only the μ-stat or m-stat was implemented (*P* < 0.001, *n* = 3). From the induction time of 72 h to the end, the co-stat process indicated constant AOX activity with an average value of 23.8 ± 0.7 U/g, however, 5.8, 4.6, and 3.6 U/g decreases were observed during μ-stat, m-stat, and DO-stat, respectively. The highest AOX activity resulted in the more efficient methanol metabolism.

Dehydrogenation of NAD^+^-dependent FLD and FDH are essential for energy generation and protein biosynthesis in *P. pastoris* (Cámara et al., 2019; Theron et al., 2019). Intracellular FLD and FDH activities are shown in Figures 5B,C. The highest FLD activity reached ∼167.5 U/g when using the co-stat or μ-stat strategy (at 36 h), which was 70.1 and 34.2 U/g higher than the maximums observed at the DO-stat and m-stat, respectively. Moreover, the co-stat process demonstrated the highest FDH activity of 154.8 U/g (at 48 h). By comparison, the lowest FLD activity was observed at the entire DO-stat process with an average value of 88.1 ± 11.5 U/g, which was 64.1 U/g lower than that of the co-stat strategy. During the last 84 h induction period, the only implementation of μ-stat strategy resulted in a decrease of 45.0 U/g in FLD activity and, a decrease of 61.5 U/g in FDH activity. Compared with co-stat process, 43.3 and 52.5 U/g lower of FLD and FDH activities were, respectively, observed, when only the m-stat strategy was used. Therefore, more efficient metabolism of the intermediates led to sufficient energy generation for *P. pastoris* cellular activities and exogenous FBG1 synthesis at the co-stat process.

Figure 5D shows the concentrations of formaldehyde. DO-stat process demonstrated 1.1 mM formaldehyde at the induction time of 12 h, which was over 3-fold higher than that observed at the co-stat process (*P* < 0.001, *n* = 3). Followed by 36 h induction by co-stat strategy, the formaldehyde concentration remained at almost constant level with an average value of 1.1 ± 0.4 mM, whereas 12.1, 4.3, and 2.0 mM higher concentrations (at 48 h) were observed at the processes of DO-stat, m-stat and μ-stat, respectively. During the last 48 h induction period, DO-stat strategy resulted in continuous accumulation of formaldehyde and reached the largest concentration of 19.5 mM. High formaldehyde concentrations were also observed when only the μ-stat or m-stat strategy was implemented, and the maximums were up to 10.2 and 11.4 mM, respectively. However, the co-stat process always demonstrated constant formaldehyde level with an average value of 1.7 ± 0.9 mM during the entire induction period. Using the DO-stat, μ-stat and m-stat strategies, higher accumulation of toxic formaldehyde in cells undermined the cell survival environment. Due to immediate utilization of formaldehyde, enhanced FBG1 production was obtained at the co-stat process.

## Discussion

In our previous studies, the glycoside hydrolase FBG1 was purified from *Fusarium* sp. CPCC 400709. FBG1 can hydrolyze trillin and produce diosgenin. To move forward to industrial production of diosgenin by biotransformation instead of acid hydrolysis, large quantities of active FBG1 were needed for clean and acid-free preparation of diosgenin. *E. coli* was implemented previously to express FBG1, but *E. coli* produced FBG1 was primary stored in cell debris in the form of inclusion body. Therefore, the exploration of other expression system was subsequently followed.

*P. pastoris* has raised an increasing interest for production of recombinant proteins (Gasser et al., 2013; Zhu et al., 2019), and so far large numbers of proteins have been expressed successfully in *P. pastoris* (Bill et al., 2011; Ahmad et al., 2014; Lee et al., 2016; Karbalaei et al., 2020). Due to the particular advantages of *P. pastoris*, this expression system was implemented to produce FBG1 for pharmaceutical diosgenin production. The production of FBG1 in *P. pastoris* was not improved through gene codon optimization. Random mutation, site directed mutation, *L*-alanine scanning and saturation mutagenesis might be alternative options. We found that the FBG1 production was affected significantly by the promotors and host strains (date not shown). GS115 strain with promotor *AOX1* (Mut^+^ type) represented the best FBG1 expression, while constitutive expression with promotor *GAP* (glyceraldehyde-3-phosphate dehydrogenase) gave much less FBG1. There was no significant difference in FBG1 production was observed between intracellular expression (without α-factor) and secreted expression (with α-factor). *C*-terminal His tag did not affect FBG1 expression in *P. pastoris*. Then, the recombinant FBG1 was expressed successfully in *P. pastoris* GS115 by transformation with the plasmid pPIC3.5K harboring *Fbg1* gene. Accordingly, high-cell density fermentation of *P. pastoris* was implemented to produce active FBG1. We investigated the effect of three conventional strategies on FBG1 expression. Furthermore, a new combined strategy (co-stat) was developed. As summarized in Table 2, various induction strategies were explored in terms of the cell growth, methanol consumption, FBG1 production and methanol metabolism. To our best knowledge, there are no open publications associated with the comparisons of strategies for enhanced production of a recombinant glucosidase used to produce diosgenin. This study represents the highest production of FBG1 in *P. pastoris*, and resulting recombinant enzyme will be subjected to biocatalysis of trillin for diosgenin production.

**TABLE 2:** *P. pastoris* cell growth, methanol consumption, FBG1 production, formaldehyde concentration and, specific activity of enzyme related to methanol metabolism during four processes.

**Fermentation strategies**			**DO-stat**	**μ-stat**	**m-stat**	**co-stat**
*P. pastoris* cell growth	Ave. specific cell growth rate (μ, h^–1^)		0.0056 ± 0.0064	0.0055 ± 0.0086	0.0055 ± 0.0054	0.0065 ± 0.0079
	Max. specific cell growth rate (μ, h^–1^)		0.020 ± 0.002 (24 h)	0.026 ± 0.001 (24 h)	0.016 ± 0.001 (48 h)	0.026 ± 0.003 (24 h)
	Max. OD_600_		405 ± 4 (96 h)	368 ± 6 (60 h)	390 ± 8 (96 h)	397 ± 6 (108 h)
Methanol consumption	Max. methanol concentration (g/L)		2.7 ± 0.06 (108 h)	4.1 ± 0.09 (108 h)	2	2 (40 h∼108 h)
	Ave. specific methanol consumption rate (g/g/h)		0.084 ± 0.020	0.061 ± 0.013	0.069 ± 0.017	0.065 ± 0.009
	Max. specific methanol consumption rate (g/g/h)		0.133 ± 0.003 (12 h)	0.086 ± 0.001 (108 h)	0.110 ± 0.002 (12 h)	0.075 ± 0.001 (96 h)
FBG1 production	Max. volumetric activity (× 10^4^, U/L)		47.3 ± 1.9 (72 h)	56.7 ± 1.2 (96 h)	66.3 ± 0.9 (108 h)	89.4 ± 1.4 (108 h)
	Max. specific activity (× 10^2^, U/g)		48.9 ± 1.2 (72 h)	64.5 ± 1.9 (96 h)	69.6 ± 1.5 (108 h)	90.0 ± 2.1 (108 h)
	Max. volumetric production (mg/L)		220.0 ± 1.4 (72 h)	249.8 ± 2.0 (96 h)	312.6 ± 1.7 (96 h)	403.3 ± 1.9 (108 h)
	Max. specific production (mg/g)		2.3 ± 0.1 (72 h)	2.8 ± 0.2 (96 h)	3.2 ± 0.2 (96 h)	4.1 ± 0.2 (108 h)
	Ave. specific production rate (mg/g/h)		0.026 ± 0.007	0.027 ± 0.007	0.034 ± 0.008	0.034 ± 0.004
	Max. specific production rate (mg/g/h)		0.036 ± 0.003 (60 h)	0.037 ± 0.002 (72 h)	0.043 ± 0.003 (36 h)	0.050 ± 0.003 (72 h)
Metabolite	Formaldehyde (CH_2_O)	Max. value (mM/g-cell)	19.5 ± 0.3 (108 h)	10.2 ± 0.2 (108 h)	11.4 ± 0.4 (108 h)	3.0 ± 0.1 (108 h)
		Ave. value (mM/g-cell)	11.9 ± 5.9	4.8 ± 3.8	5.5 ± 3.6	1.7 ± 0.9
Enzymes	Alcohol oxidase (AOX)	Max. value (U/g-cell)	35.4 ± 0.4 (36 h)	37.2 ± 0.4 (36 h)	30.6 ± 0.3 (24 h)	37.1 ± 0.2 (36 h)
		Ave. value (U/g-cell)	25.4 ± 5.4	18.8 ± 9.8	18.4 ± 5.0	24.9 ± 6.7
	Formaldehyde dehydrogenase (FLD)	Max. value (U/g-cell)	97.4 ± 0.3 (24 h)	168.3 ± 0.3 (36 h)	133.3 ± 0.2 (96 h)	167.1 ± 0.4 (36 h)
		Ave. value (U/g-cell)	88.1 ± 11.5	130.5 ± 24.0	122.0 ± 13.9	152.2 ± 20.8
	Formate dehydrogenase (FDH)	Max. value (U/g-cell)	146.3 ± 1.5 (60 h)	143.1 ± 1.4 (48 h)	102.3 ± 1.7 (72 h)	154.8 ± 1.3 (48 h)
		Ave. value (U/g-cell)	121.4 ± 33.7	103.8 ± 33.4	83.8 ± 13.5	128.1 ± 31.7

*Results reflect the maximal and average values during entire 108 h induction. Experiments were done in triplicates. Three parallel measurements for each experiment were assayed. Values are indicated by means ± S.D. Ave. indicates average value of the entire induction period. Max. indicates maximal value.*

Optimization of induction DO levels dramatically improved the production of recombinant proteins in *P. pastoris* (Pollard et al., 2007; Potvin et al., 2012; Wang et al., 2012). Maximum volumetric enzyme activities of 1.9 × 10^4^ and 1.2 × 10^4^ U/L were obtained at 3 and 40% of DO, respectively (Lee et al., 2003; Pérez-Martínez et al., 2014), which agreed with our results that 5% DO resulted in high-level expression of recombinant LXYL-P1-2 in *P. pastoris* GS115 with the maximal volumetric production of ∼900 mg/L from 1,000-L fermenter (Liu et al., 2016). The elastase inhibiting peptide expression titre increased 3-fold to 846 mg/L on average when DO set point was raised to 30% (Lee et al., 2003). Obviously, the suitable DO for different proteins need to be pre-optimized. We found that 25% DO gave the highest FBG1 production, but much less production was observed when DO was raised to 50%, which might be resulted from the cytotoxic of high oxygen (Chung, 2000). When DO-stat strategy (25% DO) was employed, methanol was utilized as we expected. The residual methanol concentrations in fermentation broth were less than 2.7 g/L, which was in the acceptable ranges for *P. pastoris* (∼4 g/L) (Zhang et al., 2000; Çelik and Çalık, 2012). However, after 72 h induction of DO-stat strategy resulted in the lowest β-glucosidase activity with the lowest FBG1 production.

It is well recognized that the methanol metabolism, metabolites, and NAD^+^-dependent dehydrogenase dramatically affect *P. pastoris* biomass accumulation and protein biosynthesis (Gao et al., 2015). Therefore, the concentrations of formaldehyde, and the enzymatic activities of AOX, FLD, FDH were measured. Results showed that formaldehyde concentrations during the DO-stat process demonstrated the highest values among all processes and almost in accordance with reported values (Gao et al., 2015), which might lead to toxicity to *Pichia* cells because of the accumulation of formaldehyde (Jungo et al., 2007; Maghsoudi et al., 2012). It’s further indicated that high concentrations of formaldehyde undermined the FBG1 production, at least in part. At DO-stat process, methanol was metabolized normally by AOX, which was consistent with methanol concentrations in the culture. However, the lowest FLD activity were observed at this process, which caused less reductive power NADH and ATP. The energy generated from formaldehyde dissimilation is particularly essential for energizing cells growth and proteins synthesis. We assumed that the methanol dissimilation pathway might be weakened, which led to insufficient energy for cell growth and proteins synthesis. Accordingly, expression of FBG1 was negatively affected. Due to poor production at DO-stat strategy, the following studies concentrated on additional strategies were further performed.

The μ-stat strategy is often used for high-cell density fermentation of *P. pastoris* (Yang and Zhang, 2017). By controlling the exponential feeding rate, μ-stat strategy is implemented in this study. Using pre-optimized exponential feeding rate (0.015 h^−l^), more FBG1 and improved β-glucosidase activity were obtained at μ*-*stat process when comparing with DO-stat strategy. Unexpectedly, cell growth was affected negatively, and a significant decrease in biomass was observed during the last 48 h induction period. Meanwhile, the experimental specific cell growth rate was varied notably although it was theoretically maintained at a constant set-value. Due to changes on the methanol consumption during induction period, over 4 g/L accumulated methanol was detected in the fermentation broth at the induction time of 108 h. Furthermore, significantly weakened activities of AOX and FDH were also observed during the last 60 h induction period. This implies that methanol was not properly metabolized at μ*-*stat process. Not only may an excessive accumulation of methanol occur when μ*-*stat strategy was implemented, but also insufficient reductive power NADH and ATP generated due to weakened FDH activity. As a result, unexpected cell growth happened, and undermined FBG1 expression was observed at μ*-*stat process. In general, methanol concentration is supposed to be close to zero at the μ*-*stat strategy. However, the methanol concentration in the bioreactor is not controlled, although its concentrations are often monitored on-line or measured off-line. Therefore, the μ-stat strategy carries the risk of undesired methanol accumulation.

Then, the m-stat strategy was subsequently investigated. The residual methanol in the fermentation broth was pre-optimized under three levels. At 2 g/L of fixed methanol concentration, further enhanced FBG1 production was obtained. After 60 h induction at m-stat strategy, over 90 g/L dry cell weight was obtained. Then, the biomass kept at constant values, which was similar to that observed at the DO-stat strategy. By comparison, less formaldehyde was formatted during the entire m-stat induction period, which was equivalent to that obtained under μ*-*stat strategy. However, the activities of AOX and FDH were lowered, which led to weakened methanol mentalism and ATP regeneration. Therefore, m-stat strategy still cannot meet the practical purposes despite the fact that the β-glucosidase activity and production of FBG1 increased dramatically during the initial 60 h induction period.

**FIGURE 6:**
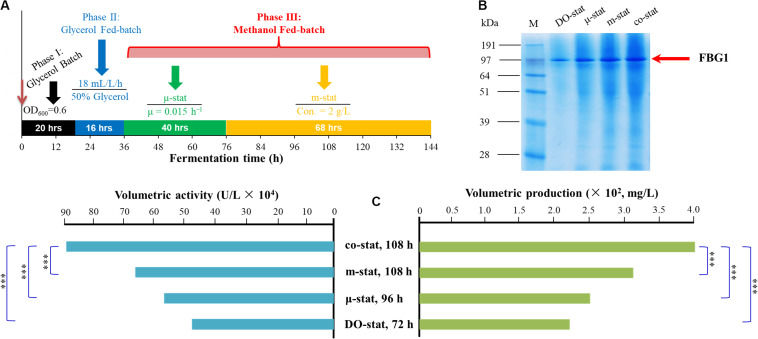
Development of co-stat strategy and FBG1 expression at various induction strategies. **(A)** Fed-batch high-cell density fermentation process of co-stat strategy. **(B)** SDS-PAGE analysis. **(C)** FBG1 expression at DO-stat, μ-stat, m-stat and co-stat processes. Bands on SDS-PAGE represent the time points of FBG1 production maximums for each strategy. Twenty microliter samples were loaded into each lane. Arrow indicates the band of recombinant FBG1. Lane M, protein molecular marker. FBG1 expression at various strategies shows the enzyme activities and production at the peak time. Error bars show means ± S.D. of triplicate independent experiments. Asterisk indicates the two-group have significant difference (^∗∗∗^*P* < 0.001).

To resolve the above-mentioned problems, the new co-stat strategy was developed. As shown in Figure 6A, a μ*-*stat process was initially implemented until the residual methanol was up to 2 g/L, and then followed by a m*-*stat process with maintained concentration of 2 g/L. Using co-stat strategy, the maximal methanol concentration in fermentation broth was ∼2-fold lower than that observed at the μ*-*stat process. Meanwhile, the highest AOX activity was significantly higher than that observed at the m-stat process. In addition, the lowest formaldehyde accumulation was seen at co-stat process, and the values almost kept constant during the last 36 h induction period. The accumulation of methanol and toxic metabolites was reduced or even largely removed at co-stat strategy. This implies that methanol was utilized effectively by *P. pastoris* and was further transformed into other required intermediates, instead of unexpected accumulation in peroxisome. Moreover, the FLD activity was much higher than that obtained at DO-stat process. The FDH activity was also much higher than that obtained under μ*-*stat and m-stat strategies. In this case, the reductive power NADH and ATP were opportunely used for *Pichia* cells growth and FBG1 biosynthesis, which indicated that the energy regeneration was positively modified. Compared with other processes, more formaldehyde left peroxisome and dissimilated in cytoplasm at the co-stat process, which further suggests that variation on methanol metabolism mode and metabolic flux might take place. The co-stat strategy prevented to reach higher methanol accumulation in fermentation broth and higher formaldehyde concentration in *Pichia* cells. High enzyme activities of AOX, FLD, and FDH were also observed at the co-stat process. Accordingly, this combined process demonstrated the most robust performance with the highest β-glucosidase activity and the highest recombinant FBG1 production. It is further verified that regulating methanol metabolism mode and energy generation of *P. pastoris* significantly enhanced the production of recombinant FBG1. More importantly, the newly developed co-stat induction strategy may be used not only for high-cell density fermentation of GS115-3.5K-*Fbg1*-s3, but also for FBG1 production from other *Pichia* strains harboring *Fbg1* gene.

## Conclusion

To efficiently produce FBG1 for the clean production of diosgenin, FBG1 was expressed successfully in *P. pastoris*. Various conventional methanol induction strategies were investigated for enhanced high-level expression of recombinant FBG1. Additionally, a new co-stat strategy composed with μ-stat and m-stat was further developed for high-cell density fermentation of the engineered yeast GS115-3.5K-*Fbg1*-s3. Due to improved methanol utilization and metabolism at the co-stat process, large amounts of formaldehyde were released by enhanced dissimilation pathway and enhanced energy generation as well, which resulted in significantly enhanced over-expression of FBG1. The highest volumetric activity reached ∼89 × 10^4^ U/L with the highest specific activity of ∼90 × 10^2^ U/g. The specific production was 4.1 mg/g, with the highest volumetric production of 403.3 mg/L, which was 183.3, 153.5, and 90.7 mg/L higher than the production obtained at the DO-stat, μ-stat and m-stat, respectively. To our best knowledge, the current study represents the highest β-glucosidase activity and production of recombinant FBG1 in *P. pastoris*. This work provides unique information on improved production of a β-glucosidase using the *P. pastoris* GS115. It is likely that the co-stat strategy may be applicable to produce similar proteins from *P. pastoris*. As a particular biocatalyst, resulting recombinant FBG1 is able to efficiently biotransform trillin, which lays solid foundation for clean and acid-free production of diosgenin, and therefore provides sustainable process to produce diosgenin in an environmental friendly way without environmental contamination and waste water. With high eco-efficiency, cost-effectiveness and sustainability, this system is a promising process for application in industrial practice.

## Data Availability Statement

The original contributions presented in the study are publicly available. This data can be found in GenBank, under accession MT793646.

## Author Contributions

WL designed, performed the experiments, and drafted the manuscript. HX, TZ, XP, JS, and HL helped to perform the experiments. BM helped to design the study. LY designed the experiments and supervised the project. WL and LY approved the final manuscript. All authors contributed to the article and approved the submitted version.

## Conflict of Interest

The authors declare that the research was conducted in the absence of any commercial or financial relationships that could be construed as a potential conflict of interest.

## Abbreviations

AOX: alcohol oxidase
DO: dissolved oxygen
FDH: formate dehydrogenase
FLD: formaldehyde dehydrogenase
OD_600_: optical cell density at 600 nm
*p*NPG: *p*-nitrophenyl -β-D-glucopyranoside
YPD: yeast extract peptone dextrose.
