# Supplementary material for: Development of a New High-Cell Density Fermentation Strategy for Enhanced Production of a Fungus β-Glucosidase in Pichia pastoris
Source: Front Microbiol. 2020 Aug 21;11:1988. doi: 10.3389/fmicb.2020.01988 (PMC7472535; doi:10.3389/fmicb.2020.01988)
Supplement: Supplementary file 1 [file Data_Sheet_1.docx]

Supplementary Material

**Development of a new high-cell density fermentation strategy for enhanced production of a fungus *β*-glucosidase in *Pichia pastoris***

Wancang Liu^1^, Haibo Xiang^1,3^, Tao Zhang^1^, Xu Pang^2^, Jing Su^1^, Hongyu Liu^1^, Baiping Ma^2^, Liyan Yu^1*^

^1^Institute of Medicinal Biotechnology, Chinese Academy of Medical Sciences & Peking Union Medical College, 1 Tian Tan Xi Li, Beijing 100050, P. R. China.

^2^Beijing Institute of Radiation Medicine, 27 Tai Ping Road, Beijing 100850, P. R. China.

^3^State Key Laboratory of Biocatalysis and Enzyme Engineering, School of Life Sciences, Hubei University, 368 You Yi Road, Wuhan 430062, P. R. China.

Correspondence:

Prof. Dr. Liyan Yu

yly@cpcc.ac.cn

# Supplementary Data

## DNA sequence

ATGACACCCTCACACGCTGTGATACAATTCCTCTTTGCGTCTCTGGCTGTTGGCCAGCAGATCTATCTTGATGCAAAGGGTCCTACTGAGCGTCCTCAATGTAAGGCTACAAAGACACATGAGCCGAAATATACACATACACCATTCAGTTATACCCTCTCTGAGACTGTGAGATACGCTACTTCGGTGCCATCTCCCACTACAACAACTACATATGCAAATCCACCAGAGTCACTCATCTCTCTCGTCCCTTCACTCTCCTTCACAACCTGGGGAAAGTGGGATCCAAATGCCACCACAAAGGCCTCAGATACAGATGATCCTTATGGACGGGCCGCATGGACTGCTCTTTGGGAACATGCAAACCCGCCCAACTTCACTGAAACGGGAATCTTCAGCACAACTGTATCTCCCACTCCCATTCCCAGCAGTGAACTAGTCCTTCCGCCACGAGACTACTTTGGTCCTTCGGATTGCTACAATTTTCCTAAGAACTTCAGCTTCGGAGTAGCTAGCTCTGCGAGTCAAATCGAGGGCGCGACTGCGGAAGAGGGCAAGGCGCCGTCTCTCATGGATATTCTCGTTCAAGATGGTCGTGTGAAGGATTATGTTACAAATGAGCACTATTACTACTACAAGCAAGATATTGAGCGCGTTGCTGCTATGGGCGCTAAGCATTTCTCGTTCAGTATCGCGTGGACGAGAATCTTGCCGTTTGCTCTACCTGGAACACCTGTGAATCAGGAGGGTATTGATCATTATAACGATGTGATCAATTTCATCCTCGAGAAGGGCATGACTCCTGAGGTCACGCTTCTTCATTTTGATACACCTCTTCAGTTCTTTGGCAGCAATCTCACCAAGGCTGCTGATCGACCTGAGATCGGCTACGTCAACGGCGGTTATCAGAATGAGACTTTCCAAGATGCTTTCGTTCACTATGCAAAGGTTGCTATGGCGCACTACGCTGATCGCGTCCCCGTTTGGTTCACCTTCAATGAGCCTCTTCTGTATTCATACAATGCTCTTTCGATCAACAATGTGGTTAAGGCTCATGCGCGAGTTTATCACTGGTATAAGGAGGAGCTTGGTGGGAAGGGCAAGATTGCTCTCAAGTTCAACAACAACTTTGGTGTACCTCGTGATCCTAAGAGTGAAGCCGATGTCTACGCTGCGGATCACTTCAACTCTATTCAGCTGGGACCGTTCTGCAACCCGATTTACCTTGGCGAGGATTACCCTGAGTCGTTCAAGCAGACTTTTGACGATTATGTCCCGCTGAGTGAGGATGATCTCAAGTACATCGGCGGTACAGCGGATTTCCTGGGTATTGATCCATATACCGCCACAGTTATTGCACCACCAGTCCCAGACGACAAAGACAGTATCCTCGAATGCGCCTCAAACTCCTCCTCCACCTTCCGTCCCTACTGTGTCAACCAAACCACCACAACCGTAAACGGGTGGAACATCGGCTACCGCTCCCAAAGCTACGTCTACATAACCCCAACCTATCTCCGCAGCTACCTCAACTACCTGCACAACACATGGAAGACTCCCGTAGCTCTCACAGAATTCGGTTTCCCCGTTTATGCCGAAGCTGAAAAAGATCTATCTGATCAGCTTTTTGATACGCCAAGAAGTATTTACTACTTGAGTTTCTTGTCTGAGACGCTTAAGGCGATTTGGGAAGATGGTGTTGAGGTTGTGGGAGCGTATGCGTGGAGCTTTGCGGATAATTGGGAGTTTGGGGATTATGATCAGCATTTTGGAATACAGACTGTTAATAGGACGACGCAGGAGAGGAGGTATAAGAAGAGTTTCTTTGATATGGTGGATTTTATGAAGGCGCGTGGTGTGGAG

## Protein sequence

MTPSHAVIQFLFASLAVGQQIYLDAKGPTERPQCKATKTHEPKYTHTPFSYTLSETVRYATSVPSPTTTTTYANPPESLISLVPSLSFTTWGKWDPNATTKASDTDDPYGRAAWTALWEHANPPNFTETGIFSTTVSPTPIPSSELVLPPRDYFGPSDCYNFPKNFSFGVASSASQIEGATAEEGKAPSLMDILVQDGRVKDYVTNEHYYYYKQDIERVAAMGAKHFSFSIAWTRILPFALPGTPVNQEGIDHYNDVINFILEKGMTPEVTLLHFDTPLQFFGSNLTKAADRPEIGYVNGGYQNETFQDAFVHYAKVAMAHYADRVPVWFTFNEPLLYSYNALSINNVVKAHARVYHWYKEELGGKGKIALKFNNNFGVPRDPKSEADVYAADHFNSIQLGPFCNPIYLGEDYPESFKQTFDDYVPLSEDDLKYIGGTADFLGIDPYTATVIAPPVPDDKDSILECASNSSSTFRPYCVNQTTTTVNGWNIGYRSQSYVYITPTYLRSYLNYLHNTWKTPVALTEFGFPVYAEAEKDLSDQLFDTPRSIYYLSFLSETLKAIWEDGVEVVGAYAWSFADNWEFGDYDQHFGIQTVNRTTQERRYKKSFFDMVDFMKARGVE

# Supplementary Figures and Tables

## Supplementary Figures

**Supplementary Figure S1. Biotransformation of trillin**


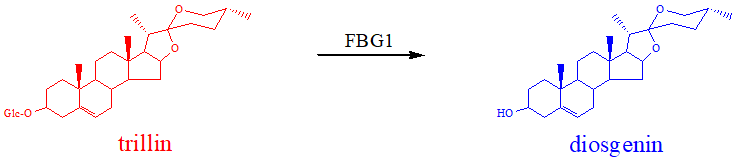


Trillin was biotransformed to diosgenin by FBG1. FBG1 was purified from *Fusarium* sp. CPCC 400709 and exogenously expressed in *P. pastoris*. Glc: glucosyl.

**Supplementary Figure S2. SDS-PAGE analysis of the recombinant FBG1 from *E. coli*.**


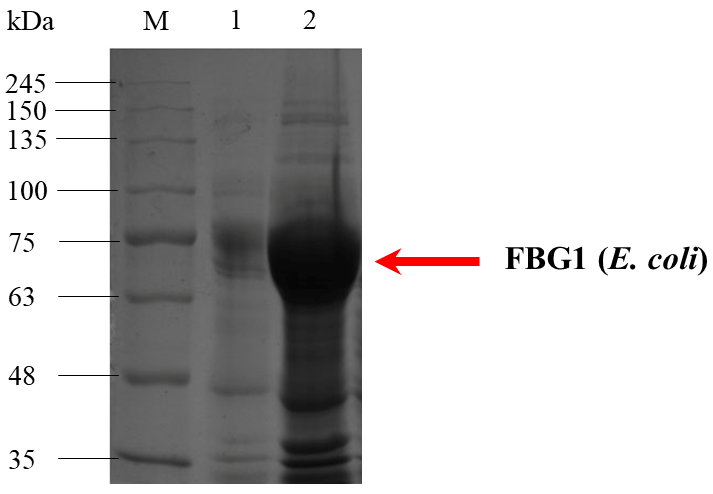


The *Fbg1* gene from CPCC 400709 was cloned into pET-30a(+), and resulting plasmid was transformed into *Escherichia coli* Transetta (DE3). The promoter was T7, and recombinant FBG1 was targeted to the cytoplasm. After induction by 0.4 mM IPTG at 20 ºC for 8 h in LB media, cell pellets from centrifugation was disrupted by sonication. After centrifugation at 4 ºC, 12,000 rpm for 15 min, the supernatant and cell debris were respectively brought to SDS-PAGE. Arrow indicates the band of the recombinant FBG1. Lanes 1 and 2 indicate supernatant and cell debris, respectively. Lane M, protein molecular marker. This protein with a predicted *N*-terminal signal sequence and few glycosylation sites was confirmed using SignalP 5.0 and expasy, respectively. There was no disulfide bond was predicted.

**Supplementary Figure S3. Simplified methanol metabolism pathway in *P. pastoris***


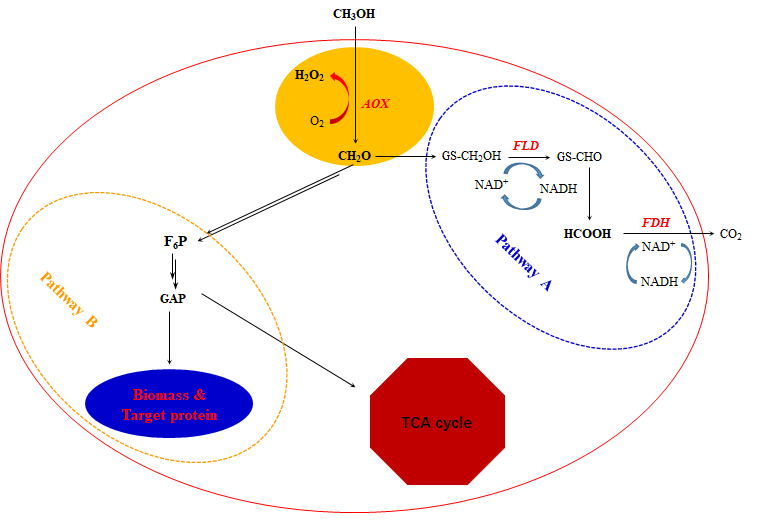


Pathway A is formaldehyde dissimilation pathway; pathway B is formaldehyde assimilation pathway. Abbreviations: AOX, alcohol oxidase; FLD, NAD^+^-dependent formaldehyde dehydrogenase; FDH, NAD^+^-dependent formate dehydrogenase; GAP: glyceraldehyde 3-phosphate; F_6_P: fructose-6-phosphate.

**Supplementary Figure S4. Construction of the engineered yeast strain and FBG1 expression in shake flasks**


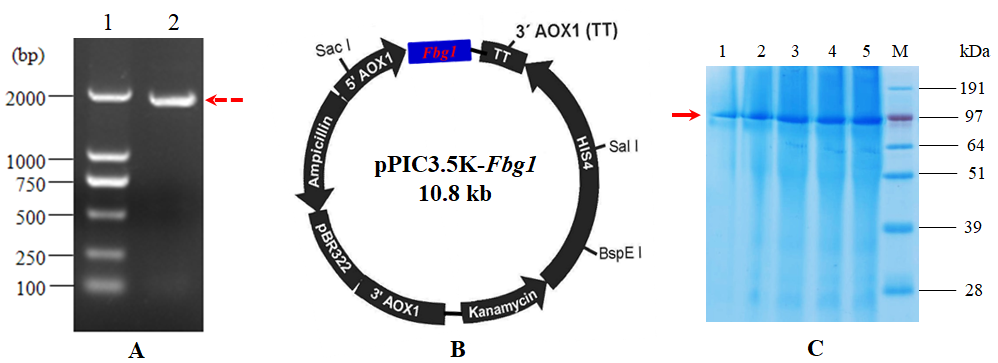


(A) PCR product of *Fbg1* gene. Lane 1 is the molecular weight ladder. Lane 2 is the band of *Fbg1* gene. (B) Scheme of the pPIC3.5K-*Fbg1* plasmid. (C) SDS-PAGE analysis of the recombinant FBG1 from shake flask. Arrow indicates the band of the recombinant FBG1. Lanes 1–5: culture supernatant of recombinant *P. pastoris* after methanol induction for 24, 48, 72, 96, and 120 h, respectively. Lane M: protein weight marker. Twenty microliter samples were loaded into each lane.

**Supplementary Figure S5. Enzymatic activity of *Pichia* *β*-glucosidases**


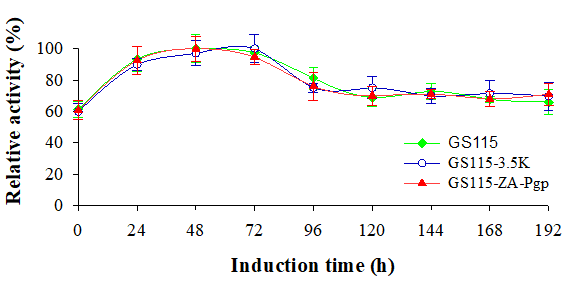


Three strains were subjected to *β*-glucosidase activity assay of *Pichia* *β*-glucosidases, mainly including the parental strain GS115 (filled green diamond), strain GS115-3.5K harboring the empty vector pPIC3.5K (open blue circle), and strain GS115-ZA-*Pgp* harboring the membrane protein P-glycoprotein (filled red triangle). Using same methods, strain GS115, GS115-3.5K and GS115-ZA-*Pgp* were cultivated in shake flasks containing BMMY medium. 1% (*v/v*) methanol was added into the culture every day at 28 ºC, 280 rpm for 192 h. Time zero indicates the initiation of methanol addition. The *β*-glucosidase activity was assayed in the same manner. The highest *β*-glucosidase activity of *Pichia* *β*-glucosidases was defined as 100% of relative activity.

**Supplementary Figure S6. Effect of sonication time on *β*-glucosidase activity of FBG1 producing *P. pastoris***


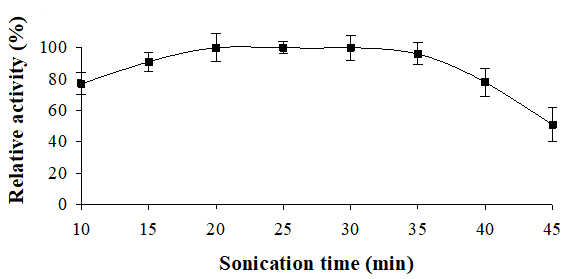


The influence of varying sonication time on the enzymatic activity of recombinant FBG1 in *P. pastoris* was determined with the sonication time ranges of 10, 15, 20, 25, 30, 35, 40, and 45 min, respectively. Using same procedures, the yeast cells were disrupted by glass beads assisted sonication under various sonication time, and resulting cell lysate was subjected to *β*-glucosidase activity assay. The highest *β*-glucosidase activity was defined as 100% of relative activity.

## Supplementary Tables

**Supplementary Table S1 Pre-optimization of DO levels, exponential feeding rates, and methanol concentrations.**

| Induction strategies | | Biomass (OD_600_) | Enzymatic activity (U/L × 10^4^) | Volumetric production (mg/L) |
| --- | --- | --- | --- | --- |
| DO-stat | 10% | 381±7 | 33.1±1.1 | 150.3±0.7 |
|  | 25% | 405±4 | 47.3±1.9 | 220.0±1.4 |
|  | 50% | 302±6 | 5.2±0.3 | 27.4±1.1 |
| μ-stat | 0.007 h^−l^ | 321±5 | 22.7±1.4 | 109.5±1.3 |
|  | 0.015 h^−l^ | 368±6 | 56.7±1.2 | 249.8±2.0 |
|  | 0.030 h^−l^ | 353±6 | 38.6±2.4 | 177.2±1.7 |
| m-stat | 0.5 g/L | 374±6 | 49.1±1.8 | 212.3±1.8 |
|  | 2.0 g/L | 390±8 | 66.3±0.9 | 312.6±1.7 |
|  | 4.5 g/L | 292±7 | 15.4±0.9 | 72.8±1.5 |

Results reflect the maximal values during the entire 108 h induction. Experiments were done in triplicates. Three parallel measurements for each experiment were assayed. Values are indicated by means ± S.D.
